# Novel Therapeutic Targets in Cutaneous Squamous Cell Carcinoma

**DOI:** 10.3389/fonc.2018.00079

**Published:** 2018-03-23

**Authors:** Teruki Yanagi, Shinya Kitamura, Hiroo Hata

**Affiliations:** Department of Dermatology, Hokkaido University Graduate School of Medicine, Sapporo, Japan

**Keywords:** cyclin-dependent kinase, mitochondria, Drp1, PD-1 antibody, epidermal growth factor receptor

## Abstract

Cutaneous squamous cell carcinoma (SCC) is one of the common cancers in Caucasians, accounting for 20–30% of cutaneous malignancies. The risk of metastasis is low in most patients; however, aggressive SCC is associated with very high mortality and morbidity. Although cutaneous SCC can be treated with surgical removal, radiation and chemotherapy singly or in combination, the prognosis of patients with metastatic SCC is poor. Recently, the usage of immune checkpoint blockades has come under consideration. To develop effective therapies that are less toxic than existing ones, it is crucial to achieve a detailed characterization of the molecular mechanisms that are involved in cutaneous SCC pathogenesis and to identify new drug targets. Recent studies have identified novel molecules that are associated with SCC carcinogenesis and progression. This review focuses on recent advances in molecular studies involving SCC tumor development, as well as in new therapeutics that have become available to clinicians.

## Introduction

In light of today’s demographic aging, skin cancer is becoming more prevalent. Cutaneous squamous cell carcinoma (SCC) is one of the most common cancers in Caucasian populations, and its prevalence is increasing (1). Cutaneous SCC accounts for 20–30% of cutaneous malignancies (2, 3). The risk of metastasis is low in most patients (2); however, aggressive SCC is associated with high morbidity and mortality (4). Although cutaneous SCC can be treated with surgery, radiation, and chemotherapy singly, or in combination, the prognosis of patients with metastatic SCC is almost always poor (3, 5). Today, chemotherapy with cisplatin alone or combined with 5-FU is being conducted with positive responses (6–9). However, the National Comprehensive Cancer Network Guidelines describe the evidence regarding systemic therapies for distant metastatic cutaneous SCC as limited. Recently, clinical trials on epidermal growth factor receptor (EGFR) inhibitors and immune checkpoint blockers have shown promising results as treatments for SCC (10–12). This review focuses on recent advances in molecular studies related to SCC tumor development and on new therapeutics that have become available.

## Recent Progress in Cutaneous SCC Therapeutics

### Novel Targeted Therapies

Cutaneous SCC overexpresses EGFR; thus, EGFR is a promising target for therapies. Cetuximab, an EGFR inhibitor, has been administered to cutaneous SCC patients. In some phase II studies, there have been good responses to cetuximab in patients with locally advanced or regional SCC types (10, 13–15). However, in distant metastatic diseases, it has been reported as ineffective. Also, tyrosine kinase inhibitors have been used to disrupt EGFR pathways. Case reports on gefetinib and imatinib have described slightly positive responses in cutaneous SCC patients (16, 17). Also, a single-arm phase II clinical trial has shown gefetinib to demonstrate modest antitumor activity in metastatic or locoregionally recurrent cutaneous SCC, with limited adverse events (18). Furthermore, bortezomib, a selective inhibitor of the 26S proteasome, may have antitumor effects in cutaneous SCC, although the mechanisms have not been clarified (19).

### Biological Modifiers

30 years ago, isotretinoin was reported to have efficacy as a treatment for local advanced cutaneous SCC alone or in combination. Interferons have also been used for local cutaneous SCC. A phase II study on bio-chemotherapy with interferons, retinoids, and cisplatin showed a positive response in 67% of locally advanced SCC cases (20). However, the efficacy against metastatic cases remains unclear.

### Cytotoxic Chemotherapy

Regrettably, recent advances in cytotoxic chemotherapy have been limited. Capecitabine, an oral prodrug of 5-FU, has been used to treat cutaneous SCC (21). In head and neck SCC cases, intra-arterial chemotherapy has been conducted as a neoadjuvant therapy (22). To date, in cutaneous SCC, no obvious evidence for positive responses has been reported, even though some cases have been described in limited detail (23).

### Immune Checkpoint Inhibitors

Recently, the US FDA approved PD-1 inhibitors (immune checkpoint inhibitors) for head and neck SCCs with continued progression during or after platinum chemotherapy (24, 25). For cutaneous SCC, several case reports have shown immune checkpoint inhibitors to have promising results. Patients with advanced cutaneous SCC responded to anti-PD-1 (nivolumab and pembrolizumab), and anti-CTLA-4 (ipilimumab) agents (26–29).

### Radiation With Chemotherapies or Immunotherapies

Platinum-based chemotherapy has been combined with local radiation. Cisplatin-based chemotherapy combined with concurrent radiation showed better results than cisplatin only (13, 30). Neoadjuvant chemotherapies before radiation were also reported to have promising results (31). Recently, the abscopal effect during radiation therapy after the administration of immune checkpoint inhibitors has been spotlighted. This effect is a phenomenon in which local radiotherapy is associated with the regression of metastatic cancer at a distance from the irradiated site (32). To date, abscopal effects have been observed in melanoma patients but not in cutaneous SCC. It is not clear whether these effects will occur in cutaneous SCCs; however, combined therapies of immune checkpoint inhibitors and radiation might have a synergistic effect.

### Other Candidates

#### Human Papilloma Virus (HPV) in Cancer Cells

Until recently, the role of HPV in cutaneous SCC was not well defined. However, a meta-analysis has found evidence that HPV is associated with cutaneous SCC (33). This systematic review indicated that cutaneous SCC harbors HPV more than normal skin does. Furthermore, an increase in HPV prevalence has been observed in SCC tumors from immunosuppressive patients. A study using an animal model showed that the interaction between UVB and HPV infection strongly promotes the development of cutaneous SCC (34). Furthermore, several targeted therapies for HPV-associated head and neck SCC have been tried (35); thus, HPV might be a promising target for cutaneous SCC as well.

#### MicroRNAs (miRs) in Cancer Cells

MicroRNAs are short, non-coding RNAs that suppress the expression of target genes. miRs can regulate various gene targets, and they play a crucial role in biological mechanisms (36). Certain miRs are associated with the onset and progression of cancers, suggesting that miRs could be targets for cancer therapies. In cutaneous SCC, several miRs are reported to be overexpressed or downregulated (36). miR-21 and miR-31 are upregulated in cutaneous SCC. The targets of these miRs are PDCD4/GRHL3/PTEN and RhoTBT1, respectively (36, 37). To inhibit the undesirable effects of up-regulated miRs, the administration of complementary nucleic acids might be a potential cancer therapy. By contrast, miR-1, miR-34a, and miR-124 are downregulated in cutaneous SCC (36). These miRs target important molecules of cell proliferation, which tend to be activated in cancer cells. Thus, promoting the over-expression of these miRs could be an option for cancer therapies. Furthermore, various miR delivery systems have been developed. Cheng et al. reported on pHLIP-mediated miR delivery methods, in which miRs could be transported across plasma membranes under the acidic conditions found in solid tumors (38). As such, miRs are also promising targets for cutaneous SCC as well as other tumors.

#### Cyclin-Dependent Kinase (Cdk) 16 in Cancer Cells

Recently, Cdk4/Cdk6 inhibitor (palbociclib) showed promising results for metastatic breast cancer (39, 40). Some Cdks are overexpressed in cutaneous SCC; thus, Cdk inhibitors may become a novel therapy option. Among Cdks, we have focused on cyclin-dependent kinase 16 (Cdk16) (also known as PCTK1, PCTAIRE1) and investigated its molecular functions. Cdk16 is a member of the Cdk family (41). Molecular functions for CDK16 are reported to vesicular transport (42) and spermatogenesis (43).

To investigate the role of Cdk16 in cancerous cells, we performed gene-knockdown experiments targeting Cdk16 (44–47). In cell lines of cutaneous SCC, prostate cancer, breast cancer, cervical cancer, and melanoma, knockdown of *Cdk16* inhibited cancer cell proliferation, and induced apoptosis over time. But, no role for Cdk16 was observed in the proliferation of non-transformed cells (IMR-90 and HaCaT cells). To identify target molecules of Cdk16, we performed yeast two-hybrid screens with human Cdk16 protein as bait. We identified tumor suppressor p27 as a Cdk16 interactor and demonstrated that Cdk16 phosphorylates p27 at Ser10 by *in vitro* kinase assays (46). The knockdown of Cdk16 modulated p27 (Ser10) phosphorylation, leading to p27 accumulation in cancerous cells. In tumor xenografts of cutaneous SCC cells, the inducible conditional knockdown of Cdk16 suppressed tumor growth (47).

To evaluate the clinical importance of Cdk16, we also studied primary tumor samples In primary tumors from the patients with breast, prostate, cutaneous basal, or SCCs, Cdk16 was expressed more highly in cancer lesions than in normal tissues (46–48). In prostate cancers, a comparison of Cdk16 immunostaining with Gleason grade revealed lower expression levels in well-differentiated tumors than in less- differentiated tumors (46). In breast cancers, Cdk16 expression was elevated in *in situ* carcinomas and invasive cancers relative to the expression in normal mammary epithelium. The significantly higher levels of Cdk16 protein that are seen in invasive cancers are associated with higher histologic grades (46). Moreover, we showed that gene knockdown of *Cdk16* sensitizes cancer cells to TNF-family cytokines, such as Fas-ligand and TNF-related apoptosis-inducing ligand (49).

To advance *in vitro* results on Cdk16 silencing, we investigated the *in vivo* therapeutic potential by using siRNA encapsulated with lipid nanoparticles (LNP) (50). Therapy of Cdk16 siRNA was performed using colorectal cancer HCT116 cells and melanoma A2058 cells. Treatment with Cdk16 siRNA-LNP reduced tumor volume and weight significantly. TUNEL staining showed increased apoptosis of cancer cells treated with Cdk16 siRNA.

**Figure 1:**
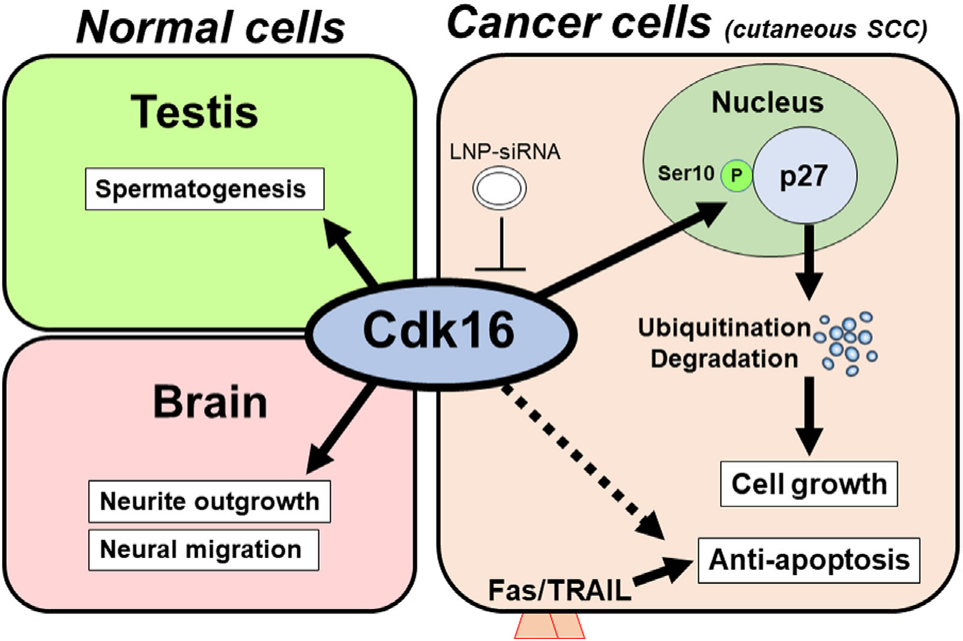
Model of the tumorigenic role of cyclin-dependent kinase 16 (Cdk16). In normal tissue (left), Cdk16 is required for spermatogenesis and neuron differentiation. In cancer cells, including cutaneous squamous cell carcinoma (SCC) cells (right), Cdk16 phosphorylates p27 at Ser10, thereby promoting p27 ubiquitination/degradation, which leads to cell cycle progression and decreased levels of apoptosis. An unknown mechanism may also exist in the Cdk16–apoptosis pathway. Lipid nanoparticle-mediated siRNA (LNP-siRNA) therapy against Cdk16 recently succeeded in a murine xenograft model.

These findings show an expected role for Cdk16 in regulating p27 expression and tumor proliferation (Figure 1). We observed these functions for Cdk16 in various cancer cells (cutaneous SCCs; basal cell carcinomas; prostate, breast, and cervical cancers; and melanomas). This implies that the p27 regulation by Cdk16 is a common machinery in human cancers.

#### Dynamin-Related Protein 1 (Drp1) in Cancer Cells

**Figure 2:**
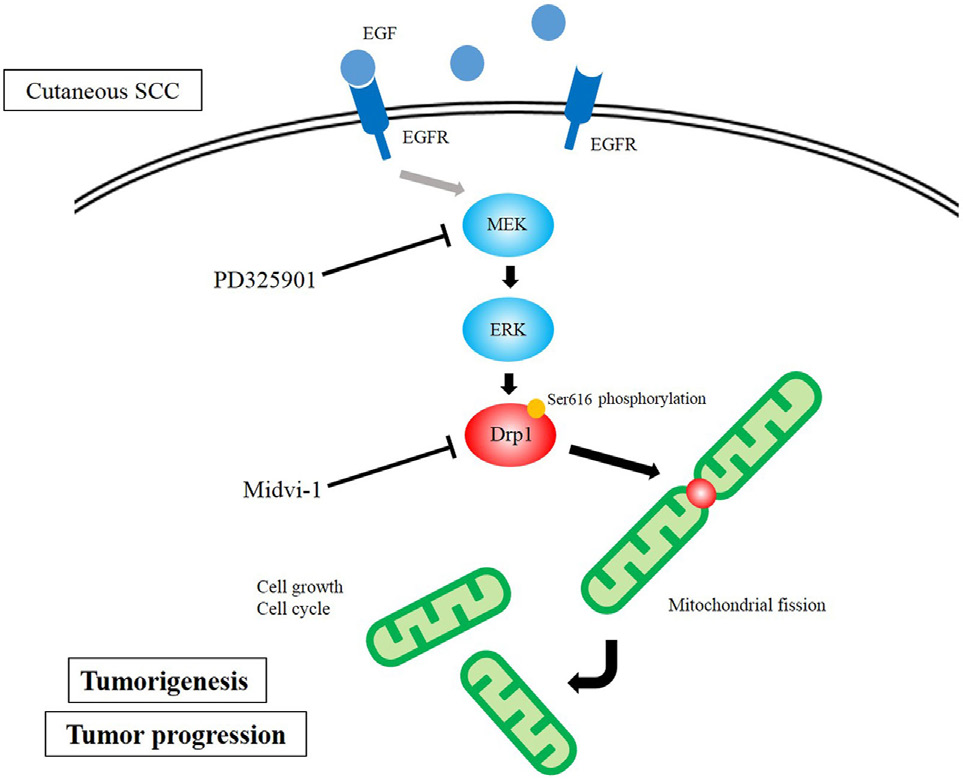
Diagram of dynamin-related protein 1 (Drp1) function in cutaneous squamous cell carcinoma (SCC) cells. MAPK signaling activates Drp1 *via* the phosphorylation of Drp1. The overexpression of Drp1 induces mitochondrial fission, which results in cell growth and assists cell cycle.

We have also focused on the mitochondria-associated molecule Drp1 (51). Drp1 regulates mitochondrial fission. Recently, it was found to be associated with cancer cell proliferation in melanoma and lung cancer (52, 53). Disrupted mitochondrial networks induce cell cycle arrest and apoptosis (53, 54). Also, Drp1 has been reported as a prognostic factor in several malignancies, such as lung adenocarcinomas and glioblastomas (55, 56). Based on these previous studies, we investigated the role of Drp1 in cutaneous SCCs. Drp1 gene-knockdown SCC cells showed lower cell proliferation than control cells, as assessed by cell counting and clonogenic assays. DNA content Cell Cycle analysis showed Drp1 knockdown to cause G2/M phase arrest. Morphologically, the depletion of Drp1 resulted in an elongated mitochondrial network. The MEK inhibitor, PD325901, inhibited cell proliferation, as well as inhibiting the phosphorylation of ERK1/2 and Drp1 (Ser616). PD325901 also caused the dysregulation of the mitochondrial network. In tumor xenografts of DJM1 SCC cells, the knockdown of Drp1 suppressed tumor growth *in vivo*. Clinically, the expression levels of Drp1 were higher in cutaneous SCC specimens than in normal epidermis, and those levels correlated positively with advanced clinical stages. Our data reveal a pivotal function for Drp1 in mediating tumor growth, mitochondrial fission, and cell cycle in cutaneous SCCs (Figure 2), suggesting that Drp1 could be a novel target for cutaneous SCC therapies.

## Concluding Remarks

In the past 10 years, novel therapeutic agents for cutaneous SCC have been developed. EGFR inhibitors and immune checkpoint inhibitors have shown particularly promising results. Furthermore, these novel treatments can be used a monotherapies or in combination with radiation; thus dermatologists and oncologists will be able to choose better treatments depending on conditions of the patient and the stage of the disease. Also, novel targeting molecules and inhibitors have been developed.

## Author Contributions

TY and HH designed the study. TY and SK wrote the paper. HH supervised the study.

## Conflict of Interest Statement

The authors declare that the research was conducted in the absence of any commercial or financial relationships that could be construed as a potential conflict of interest.
